# Exosomal lncRNA PCAT1 Promotes Tumor Circulating Cell-Mediated Colorectal Cancer Liver Metastasis by Regulating the Activity of the miR-329-3p/Netrin-1-CD146 Complex

**DOI:** 10.1155/2022/9916228

**Published:** 2022-08-31

**Authors:** Xingbao Fang, Yongping Xu, Kezhi Li, Peiwan Liu, Hong Zhang, Yang Jiang, Jianwei Tang, Yuehong Li

**Affiliations:** Department of Hepatobiliary Pancreatic Surgery, The First People's Hospital of Qujing City, Yunnan Province (Qujing Hospital Affiliated to Kunming Medical University), Qujing 655000, China

## Abstract

**Objective:**

This study explored the colorectal cancer exosome lncRNA prostate cancer associated transcript 1– (PCAT1) mediated circulating tumors and the mechanism of cell colorectal cancer liver metastasis.

**Methods:**

Exosomes were extracted from the primary colorectal cancer (CRC) cell lines HCT116 and SW480 and cultured with T84 and human umbilical vein endothelial (HUVE) cells. The expression of PCAT1 and miR-329-3p was detected by real-time quantitative polymerase chain reaction (RT-qPCR), the expression of Netrin-1, CD146, and epithelial mesenchymal transition (EMT) related proteins was detected by Western blot, the proliferation activity of T84 cells was detected by cell counting kit 8 (CCK-8), and cell migration was detected by Transwell. The expression of the F-actin signal was detected by immunofluorescence after coculture of exosomes with human umbilical vein endothelial cells (HUVECs). Changes in subcutaneous tumor and liver nodule size after PCAT1 deletion were observed in a mouse model of liver metastasis from rectal cancer.

**Results:**

PCAT1 expression was upregulated in primary cell lines and their exosomes. After exosomes were cocultured with colorectal cancer tumor circulating T84 cells, the expression of Netrin-1 and CD146 was upregulated, the expression of miR-329-3p was downregulated, the proliferation and migration ability of T84 cells were enhanced, and EMT occurred. After knocking down PCAT1, the above phenomenon was reversed. Similarly, after exosomes were cocultured with HUVECs, the expression of the F-actin signal increased, and after PCAT1 was knocked down, the F-actin signal also decreased. PCAT1 regulates miR-329-3p/Netrin-1 and affects the biological behavior of T84 and F-actin signal expression in HUVECs. In a mouse model of colorectal cancer liver metastasis, knocking down PCAT1 significantly reduced the nodules formed by liver metastasis in mice.

**Conclusions:**

LncRNA PCAT1 derived from colorectal cancer exosomes regulates the activity of the Netrin-1-CD146 complex in circulating tumor cells (CTCs) to promote the occurrence of colorectal cancer EMT and liver metastasis and provides new molecular targets for the treatment of colorectal cancer liver metastasis.

## 1. Introduction

As a common malignant tumor of the digestive system, colorectal cancer (CRC) incidence is rapidly increasing worldwide [1]. The occurrence of colorectal cancer has a wide range of effects, with 1.36 million people being affected worldwide, accounting for nearly 10% of global cancers [2]. The mortality rate is also extremely high, with approximately half a million deaths per year worldwide [3]. At present, the comprehensive treatment of colorectal cancer with surgery and the development and application of molecular targeted drug therapy have significantly improved the five-year survival rate of colorectal cancer. However, approximately 35-55% of patients with colorectal cancer still develop liver metastasis, which seriously affects the surgical efficacy and prognosis of patients with colorectal cancer [4]. Studies have reported that metastatic colon cancer significantly reduces the survival rate of patients, with the 5-year survival rate dropping from 90% to 10% [5]. The main cause of death in 90% of cancer patients is tumor metastasis, and epithelial-mesenchymal transition (EMT) is closely related to tumor metastasis [6]. The development of EMT in colorectal cancer has become a research hotspot in recent years, and the molecular mechanism is still being explored [7].

Circulating tumor cells (CTCs) are epithelial cancer cells derived from primary or metastatic tumors and are considered precursors of metastasis, associated with metastasis and associated with poor prognosis [8]. Studies have shown that CTCs are readily survivable and metastatic after epithelial-mesenchymal transformation and are therefore closely associated with a high metastatic potential in many solid cancers, including CRC [9]. In addition, CTCs are an important biomarker for the prognosis of colorectal cancer. Therefore, it is of great significance to further explore the related molecular mechanisms of CTC-mediated CRC metastasis [10]. Exosomes are microbubbles (30-100 nm) that can be produced by a variety of cell types and act as intermediaries in cell-to-cell communication [11]. Many research reports point out that the regulation of the tumor microenvironment mediated by exosomes promotes the metastasis and development of cancer cells [12, 13]. Primary tumor-derived exosomes have been found to promote tumor metastasis by regulating CTC adhesion [14]. However, the molecular mechanism by which exosomes-CTCs promote liver metastasis of colorectal cancer has not yet been reported.

Recently, lncRNAs in cancer progression have been gradually discovered [15]. For example, lncRNA HOTAIR is a marker of abnormal regulation of the lung cancer cell cycle [16]. lncRNA MALAT1 can affect the proliferation, invasion, and migration of colorectal cancer by regulating SOX9 [17]. Studies have found a variety of lncRNA disorders in colorectal cancer [18]. Several studies have reported that exosomes can mediate lncRNA prostate cancer associated transcript 1 (PCAT1) to participate in a series of biological behaviors of cancer cells [19–21]. Although abnormal expression of PCAT1 has also been found in colorectal cancer, its role in colorectal cancer remains to be further studied [22, 23]. Netrin-1 is a secreted protein associated with laminin and a neuronal axon guidance factor. On the one hand, Netrin-1 has the functions of regulating axon growth, cell migration, and angiogenesis [24]. On the other hand, Netrin-1 is also involved in the regulation of the biological behavior of tumor cells [25]. Studies have found that Netrin-1 works by binding to specific receptors. The currently known Netrin-1 receptors can be divided into two categories according to their structure, one category is the deleted in colorectal cancer (Dcc), and the other is uNC5 homolog (uNc5 homolog, uNc5H) [26]. Studies have shown that CD146 is also one of the receptors for Netrin-1, and Netrin-1 can affect downstream signaling in a CD146-dependent manner [27]. As a multifunctional molecule, CD146 has been reported to be involved in many biological processes, such as morphogenesis in tumor metastasis and tissue regeneration [28]. Other earlier studies have found that abnormal expression of CD146 is associated with colorectal cancer progression [29]. However, whether and how the Netrin-1-CD146 complex participates in EMT and induces colorectal cancer development is still unknown.

In our research, we predicted the ceRNA network of PCAT1 and Netrin-1 through biometric analysis and found that PCAT1-miR-329-3p-Netrin-1 can form a ceRNA network. miR-329-3p has been found to be negatively correlated with cancer development in a variety of cancers [30–32]. On this basis, we further confirmed that PCAT1 can affect the Netrin-1-CD146 complex by regulating miR-329-3p, thus affecting the EMT process and liver metastasis process of colorectal cancer.

## 2. Materials and Methods

### 2.1. Cell Culture and Transfection

Human umbilical vein endothelial cells (HUVECs), human normal colon NCM460 cells, and human colorectal cancer HCT116, SW480, and T84 cells were cultured in Dulbecco's modified Eagle's medium (DMEM) containing 10% fetal bovine serum (FBS) (HyClone, Logan, UT, USA) and penicillin at 37 °C in a 5% CO_2_ incubator. When the cells reached the logarithmic growth phase, the transfection reagent lipofectamine 2000 (Invitrogen, Waltham, M A, USA) was used for transfection. After 48 hours of transfection, the cells were collected for detection in subsequent experiments.

### 2.2. Isolation and Observation of Exosomes

Exosomes were extracted according to the kit instructions: Centrifuge at 2000 × *g* at 4 °C for 20 minutes to remove residual cells, add 1/3 volume of reagent to the sample, mix the sample completely, and incubate at 4 °C overnight; centrifuge at 13,000 × *g* at 4 °C for 1 h, and carefully discard the supernatant; centrifuge at 13,000 × *g* at 4 °C for 30 s, discard the supernatant, and resuspend the exosomes with 100-200 *μ*l RNase-free water exosomes. Then, the morphology and size of exosomes were observed under a transmission electron microscope.

### 2.3. Real-Time Quantitative Polymerase Chain Reaction (RT-qPCR)

A total RNA kit was used to extract total cell RNA, and total RNA was reverse-transcribed into cDNA. Primers were designed according to the principle of primer design, cDNA was used as a template, the reaction system and conditions were set up according to the PCR kit instructions, 6 multiple wells were set up per well, real-time fluorescence quantification was used to obtain the average Ct value, and the 2^-△△Ct^ formula was used to calculate and detect mRNA. For the relative expression level, the experiment was repeated 3 times.

### 2.4. Western Blotting

Total cell protein was routinely extracted, and the protein was quantified with a bicinchoninic acid (BCA) kit. The same amount of protein (50 *μ*g) was collected from each channel, separated by 10% sodium dodecyl sulfate-polyacrylamide gel electrophoresis (SDS-PAGE), and transferred to polyvinylidene fluoride (PVDF). The PVDF membrane was blocked with 5% nonfat milk powder for 2 h, then the blocking solution was poured out, and several primary antibodies such as netrin-1, CD146, vimentin, fibronectin, N-cadherin, E-cadherin, and ZO-1 were added and incubated on a 4°C shaker. After overnight, the membrane was washed with PBS and then labeled with horseradish peroxidase-labeled (1:2000 dilution), incubated at room temperature for 1 hour, and the electrochemiluminescent solution was added after washing the membrane, and the results were observed with a gel imaging system. ImageJ software was used to analyze the gray value of each protein band.

### 2.5. CCK8 Experiment

The cells were inoculated in 96-well plates. The extracted exosomes and cells were cultured in an incubator for 24 hours, and then the cells were transfected with plasmids of different treatment groups or incubate in an empty cell incubator for 24 hours. CCK-8 solution was added, and the solution was incubated for 4 hours, and the absorbance was determined at 450 nm.

### 2.6. Immunofluorescence

Cells of different treatment groups were inoculated on coverslips, and after they were fully adhered, they were cultured by adding DMEM. Then, the cells of each treatment group were washed 3 times with phosphate-buffered saline (PBS) and fixed with 4% paraformaldehyde for 20 min and 0.2% Triton X-100. After 100 minutes of permeabilization, the cells were stained with 100 ng/ml rhodamine-phalloidin for 60 minutes, mounted with 30% glycerol-PBS, and observed for the distribution of F-actin under a fluorescence microscope.

### 2.7. Construction of Tumor Formation and Metastasis Models In Vivo

Colon cancer cell liquid was cocultured with exosomes and colon cancer cell liquid that knocked down PCAT1 under the condition of cocultivation and injected subcutaneously on both sides of the spine of nude mice near the hind legs. The tumor was measured every specified number of days. The average value of the tumor size of each group was taken. After the measurement was completed on the 28th day, the mice were sacrificed, the tumor tissues were removed and weighed, and pictures were taken. Similarly, the abovementioned cell fluid was injected into nude mice, and the nude mice were sacrificed 28 days later to observe lung metastasis.

### 2.8. Statistical Methods

All data were statistically analyzed by SPSS 17.0 software. Two independent samples were analyzed by *t*-test, and multiple group means were compared by analysis of variance (ANOVA).

## 3. Results

### 3.1. Expression of lncRNA PCAT1 in the Primary Colorectal Cancer Cell Lines HCT116 and SW480 and their Exosomes

**Figure 1:**
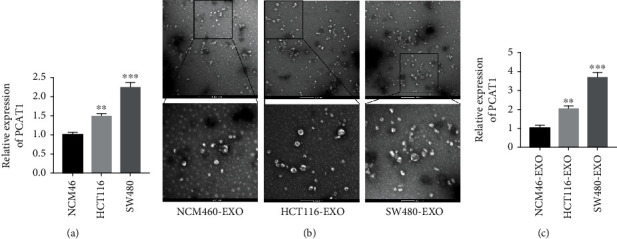
Expression of PCAT1 in colorectal cancer cells and their exosomes. (a) RT-qPCR was used to detect the expression of PCAT1 in HCT116 and SW480 cells. ^∗∗^*P* < 0.01, ^∗∗∗^*P* < 0.001*vs* NCM46. (b) The shape and size of exosomes were observed by transmission electron microscopy. (c) RT-qPCR detected the expression of PCAT1 in the exosomes. ^∗∗^*P* < 0.01, ^∗∗∗^*P* < 0.001*vs* NCM46-EXO.

We first detected the expression of PCAT1 in the primary tumor cell lines HCT116 and SW480. The RT-qPCR results showed that compared with NCM460 cells, the expression of PCAT1 in HCT116 and SW480 cells was significantly upregulated (Figure 1(a)). Exosomes of HCT116 and SW480 were extracted, and circular particles with double membranes approximately 80-100 nm in size were observed under transmission electron microscopy (TEM), which was consistent with the common size of exosomes (Figure 1(b)). Similarly, the expression of PCAT1 was upregulated in HCT116 and SW480 exosomes compared with NCM460 exosomes (Figure 1(c)).

### 3.2. Effects of Exosomal PCAT1 on the Biological Behavior of Circulating Tumor T84 Cells

**Figure 2:**
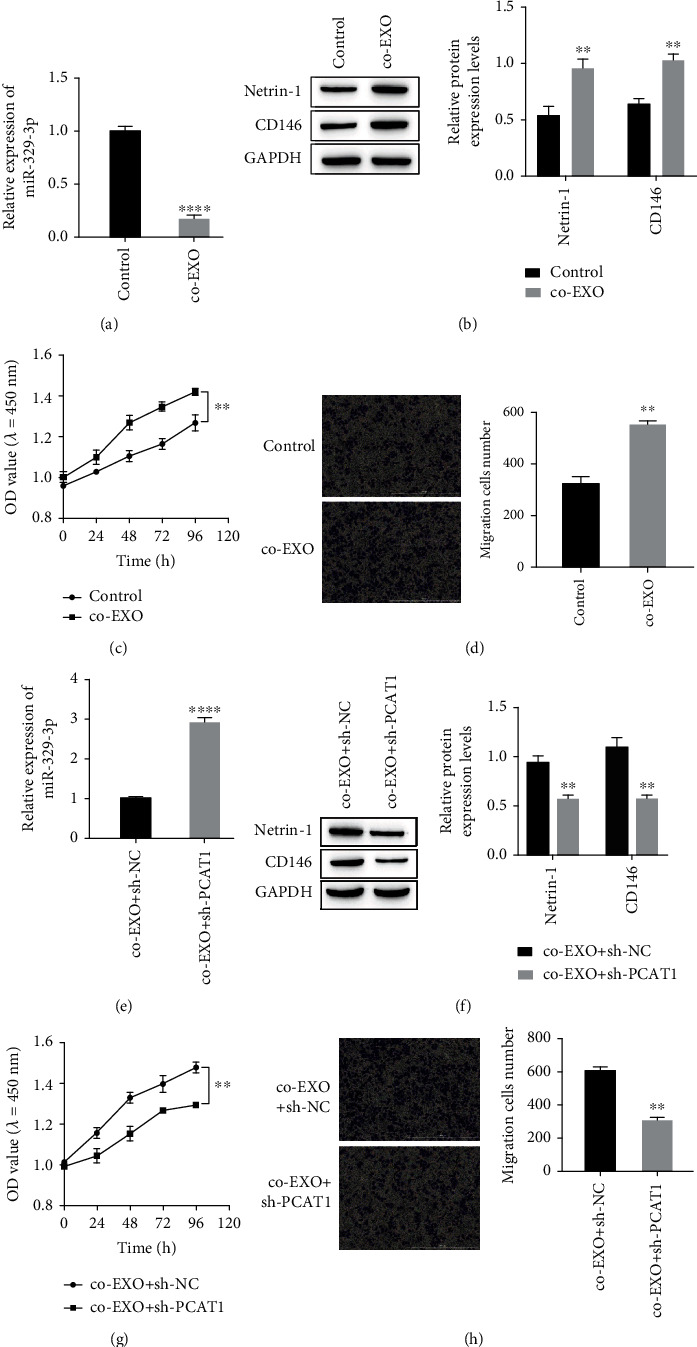
The effect of exosomal PCAT1 on the biological behavior of circulating tumor T84 cells. (a) RT-qPCR was used to detect the expressions of miR-329-3p after co-culture of exosomes with T84. (b) Western blot was used to detect the expressions of Netrin-1 and CD146 after co-culture of exosomes with T84. (c) CCK-8 detected the cell proliferation activity after co-culture of exosomes with T84. (d) Transwell detected the cell migration ability after co-culture of exosomes with T84. ^∗∗^*P* < 0.01, ^∗∗∗∗^*P* < 0.0001*vs* Control. (e) RT-qPCR. was used to detect the expressions of miR-329-3p in exosomes co-cultured with T84 after PCAT1 knockdown. (f) Western blot was used to detect the expressions of Netrin-1 and CD146 in exosomes co-cultured with T84 after PCAT1 knockdown. (g) CCK-8 detected the cell proliferation activity of exosomes and T84 co-cultured after PCAT1 knockdown. (h) Transwell detected the cell migration ability of exosomes and T84 co-cultured after PCAT1 knockdown. ^∗∗^*P* < 0.01, ^∗∗∗∗^*P* < 0.0001*vs* co-EXO+sh-NC.

To confirm that colorectal cancer exosome PCAT1 can further promote the progression of colorectal cancer by influencing the biological behavior of circulating tumor cells, we cocultured the extracted exosomes with T84 cells and found that miR-329-3p expression was significantly downregulated in the cocultured group compared with the control group (Figure 2(a)). The expression of Netrin-1 and CD146 was significantly increased (Figure 2(b)). At the same time, cell counting kit-8 (CCK-8) test results showed that the proliferation activity of T84 cells was significantly increased after coculture with exosomes (Figure 2(c)), and Transwell results showed that the migration ability of T84 cells was improved (Figure 2(d)). After knocking down PCAT1, the above phenomenon was reversed. That is, miR-329-3p expression was upregulated (Figure 2(e)), and Western blot results showed that Netrin-1 and CD146 expression were significantly decreased (Figure 2(f)). The proliferation activity and migration ability of T84 cells were significantly decreased (Figures 2(g)–2(h)).

### 3.3. Effects of Exosomal PCAT1 on the EMT Process and Fibroid Actin

**Figure 3:**
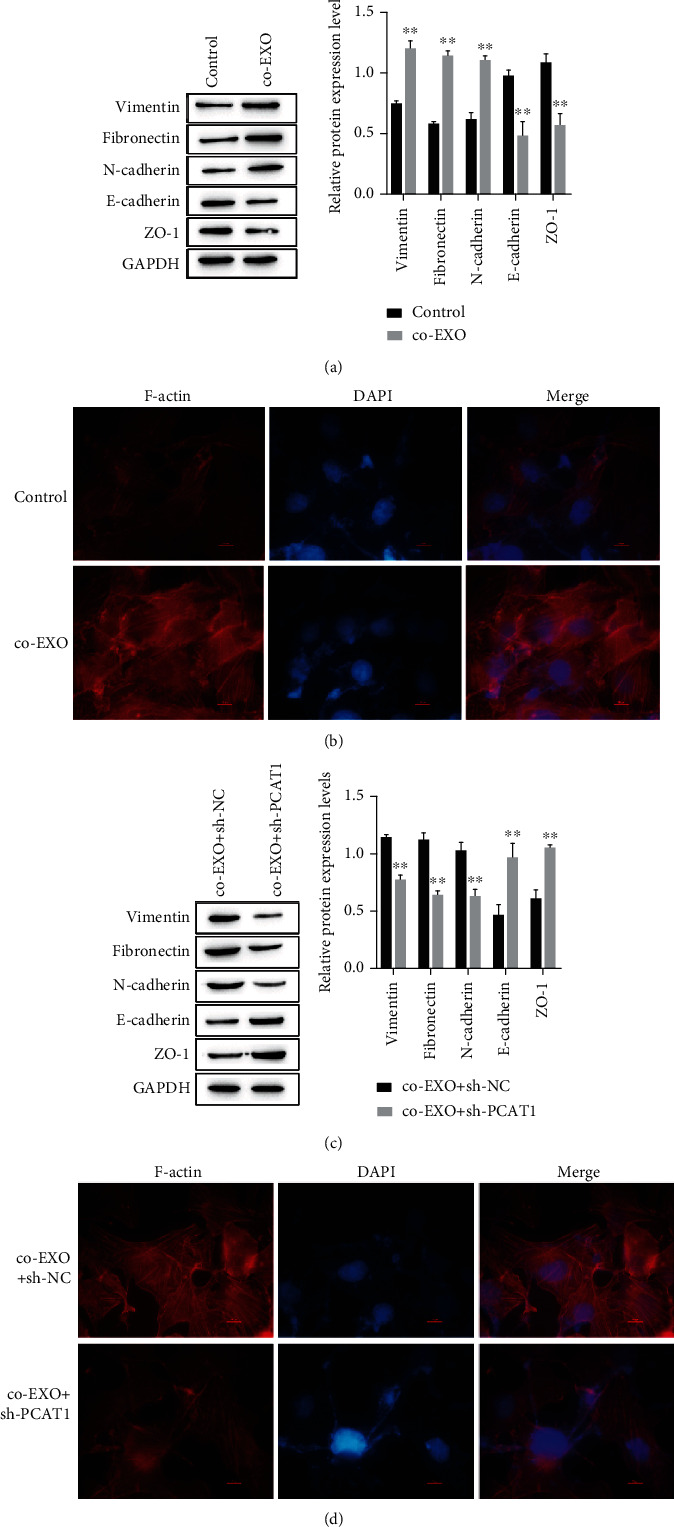
The effect of exosomal PCAT1 on the EMT process and fibrous actin. (a) Western blot was used to detect the expression of EMT-related proteins in exosomes co-cultured with T84. (b) The changes of F-actin after co-culture of exosomes with HUVECs were detected by immunofluorescence. ^∗∗^*P* < 0.01*vs* Control. (c) Western blot was used to detect the expression of EMT-related proteins in exosomes co-cultured with T84 after PCAT1 knockdown. (d) The changes of F-actin in exosomes and HUVECs co-cultured after PCAT1 knockdown were detected by immunofluorescence. ^∗∗^*P* < 0.01*vs* co-EXO+sh-NC.

The formation of fibroactin plays an important role in tumor metastasis. Therefore, exosomes were cultured with T84 cells and HUVECs, and EMT-related protein expression and fibroactin formation were detected to determine whether PCAT1 promotes colorectal cancer by affecting the EMT process and fibroactin formation. The results showed that exosomes could promote the development of EMT in T84 cells; that is, the expression levels of vimentin, fibronectin, and N-cadherin were upregulated, while the expression levels of E-cadherin and ZO-1 were downregulated (Figure 3(a)). Immunofluorescence was used to detect the F-actin signal of HUVECs, and F-actin was found to significantly coarsen and cluster into bundles, forming a large number of stress fibers around and inside cells (Figure 3(b)). When PCAT1 was knocked down, the above phenomenon was reversed. Compared with the coculture group, the expression of vimentin, fibronectin, and N-cadherin was decreased in the coculture+sh-PCAT1 group, while the expression levels of E-cadherin and ZO-1 were increased (Figure 3(c)). Immunofluorescence detection results showed that after knocking down PCAT1, F-actin gradually depolymerized, and stress fiber formation decreased (Figure 3(d)).

### 3.4. Identification of Targeted Regulatory Relationships

**Figure 4:**
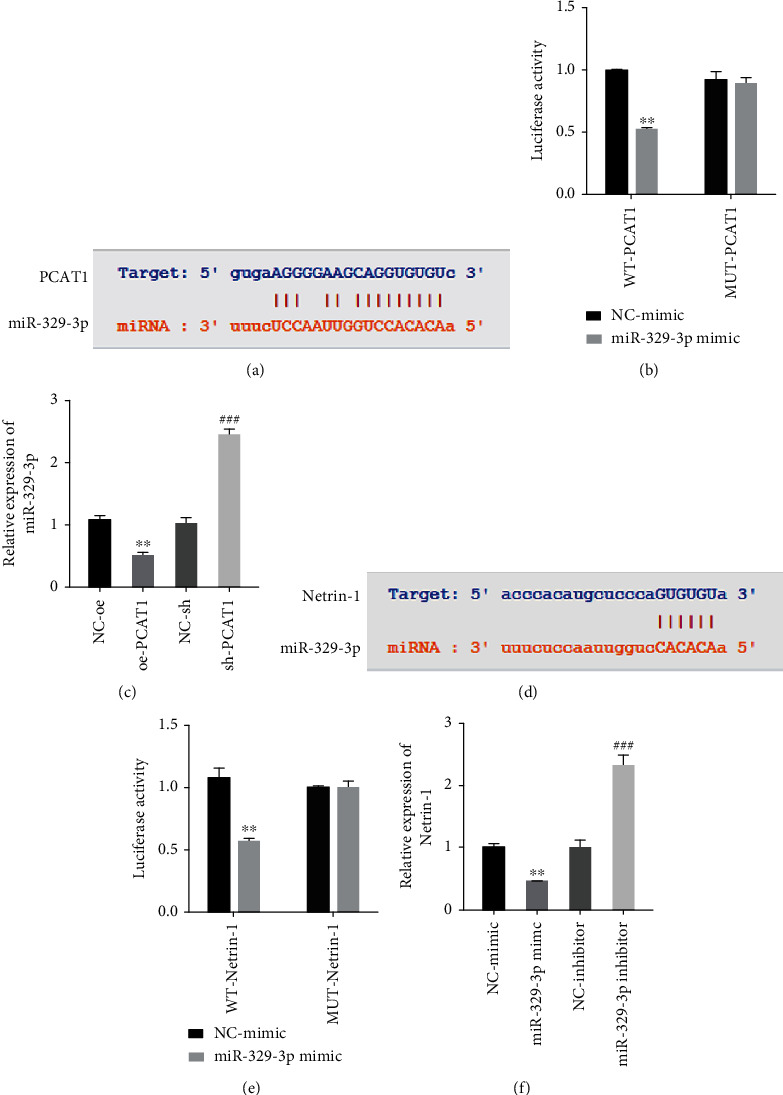
Identification of targeted regulatory relationships. (a) The StarBase website predicts the targeted sites. (b) The dual luciferase gene report experiment. ^∗∗^*P* < 0.01*vs* NC-mimic. (c) The expression of miR-329-3p was detected by RT-qPCR. ^∗∗^*P* < 0.01*vs* NC-oe; ^###^*P* < 0.001*vs* NC-sh. (d) The StarBase website predicts the targeted sites. (e) The dual luciferase gene report experiment. ^∗∗^*P* < 0.01 vs NC-mimic. (f) The expression of Netrin-1 was detected by RT-qPCR. ^∗∗^*P* < 0.01*vs* NC-mimic; ^###^*P* < 0.001*vs* NC-inhibitor, respectively.

StarBase predicted that there was a binding site between PCAT1 and miR-329-3p (Figure 4(a)). Further double luciferase gene reporter experiments showed that the relative fluorescence intensity of PCAT1 in the miR-329-3p mimic+PCAT1-3′UTR group was significantly lower than the relative fluorescence intensity in the NC-mimic+PCAT1-3′UTR WT group. However, there was no difference in fluorescence intensity between the two groups when the binding site was mutated. (Figure 4(b)). RT-qPCR detection results further confirmed that PCAT1 can target and negatively regulate miR-329-3p (Figure 4(c)). Additionally, through the StarBase prediction, we found that Netrin-1 is a downstream target of miR-329-3p (Figure 4(d)) and the targeting relationship between the two was further confirmed by a dual-luciferase gene reporting experiment (Figure 4(e)). RT-qPCR results showed that miR-329-3p could negatively regulate Netrin-1 expression (Figure 4(f)).

### 3.5. Exosomal PCAT1 Affects the Biological Behavior of T84, EMT Process and Fibroid Actin Signaling by Targeting the miR-329-3p/Netrin-1 Axis

**Figure 5:**
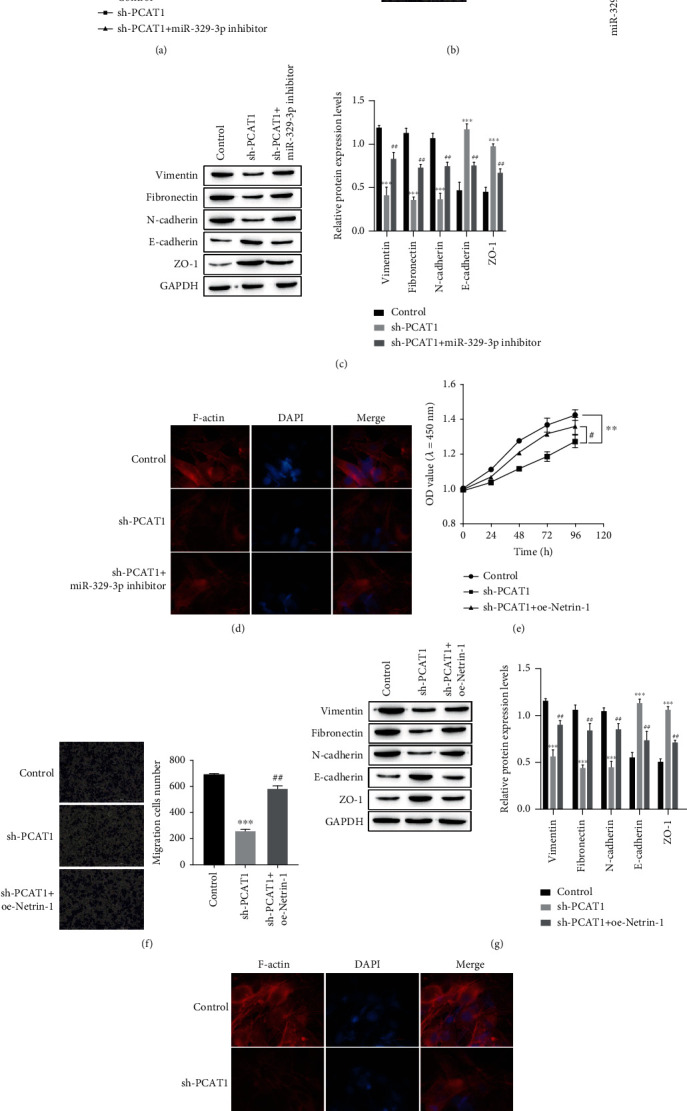
PCAT1 regulation of the miR-329-3p/Netrin-1 axis affects the biological behavior of T84 and the ability of fibroid actin to form stress fibers. (a) The proliferation activity of T84 cells was detected by CCK-8 assay. (b) Transwell assays were used to detect the migration ability of T84 cells. (c) Western blot was used to detect the expression of EMT-related proteins. (d) Stress fibers formed by F-actin were detected in HUVECs by immunofluorescence. (e) The proliferation activity of T84 cells was detected by CCK-8 assay. (f) Transwell assays were used to detect the migration ability of T84 cells. (g) Western blot to detect the expression of EMT-related proteins. (h) Stress fibers formed by F-actin were detected in HUVECs by immunofluorescence. ^∗∗^*P* < 0.01, ^∗∗∗^*P* < 0.001*vs* Control; ^#^*P* < 0.05, ^##^*P* < 0.01*vs* sh-PCAT1.

To further explore the specific molecular mechanism of colorectal cancer exosomal PCAT1 in the development of colorectal cancer, we cocultured exosomes with T84 cells and then transfected the cells with sh-PCAT1 or cotransfected them with sh-PCAT1 + miR-329-3p inhibitor or sh-PCAT1 + oe-Netrin-1. The proliferation activity, migration ability, and expression of EMT-related proteins were observed. The exosomes were then cocultured with HUVE cells, and the same transfection treatment was performed to observe the formation of F-actin in HUVE cells. CCK-8 test results showed that sh-PCAT1 transfection could effectively reduce the promoting effect of exosome coculture on T84 cell proliferation, but retransfection with miR-329-3p inhibitor could reverse this phenomenon (Figure 5(a)). The same Transwell assay and Western blot detection also showed that the transfection of sh-PCAT1 could effectively inhibit the migration of T84 cells and the occurrence of EMT, while the cotransfection of sh-PCAT1 + miR-329-3p inhibitor could reverse this phenomenon (Figures 5(b)–5(c)). The results of immunofluorescence showed that transfection of sh-PCAT1 reduced the formation of F-actin in HUVECs, while the formation of F-actin increased in the sh-PCAT1 + miR-329-3p inhibitor group compared with the sh-PCAT1 group (Figure 5(d)). In addition, the test results also showed that compared with the sh-PCAT1 group, the proliferation and migration of T84 cells in the sh-PCAT1 + oe-Netrin-1 group were enhanced (Figures 5(e)–5(f)), the expression levels of vimentin, fibronectin, and N-cadherin were upregulated, and the expression levels of E-cadherin and ZO-1 were downregulated (Figure 5(g)). Likewise, the formation of F-actin was increased in HUVE cells in the sh-PCAT1 + oe-Netrin-1 group compared with the sh-PCAT1 group (Figure 5(h)).

### 3.6. Effect of Exosome PCAT1 on Subcutaneous Tumorigenesis and Liver Metastasis of Colon Cancer In Vivo

**Figure 6:**
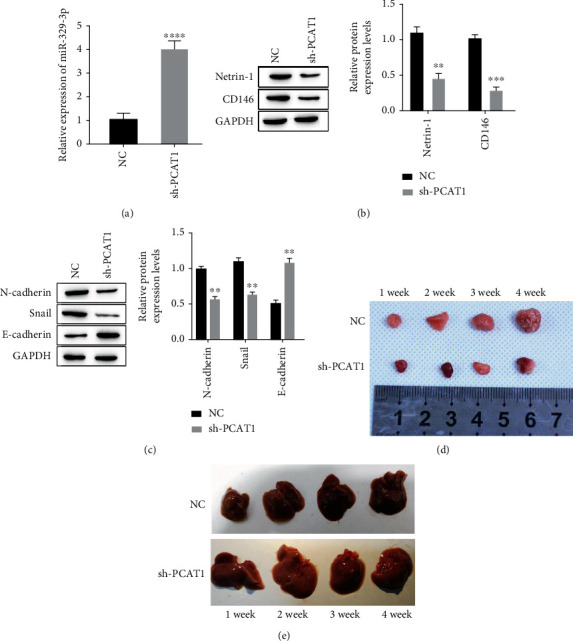
Effect of exosome PCAT1 on subcutaneous tumorigenesis and liver metastasis of colon cancer in vivo. (a) The expression of miR-329-3p was detected by RT-qPCR. (b) The expression of Netrin-1 and CD146 was detected by western blot. (c) Western blotting was used to detect the expression of EMT-related proteins. (d) Observation of subcutaneous tumorigenesis size. (e) Observation of hepatic nodules. ^∗∗^*P* < 0.01, ^∗∗∗^*P* < 0.001, ^∗∗∗∗^*P* < 0.0001*vs* NC.

To further clarify the influence of PCAT1 on tumor formation and colorectal cancer metastasis, we constructed a nude mouse model of subcutaneous implantation tumors of colon cancer and liver metastasis of colon cancer. The results of RT-qPCR showed that after knocking down PCAT1, miR-329-3p increased (Figure 6(a)). Netrin-1 and CD146 decreased (Figure 6(b)), N-cadherin and Snail decreased, and E-cadherin increased (Figure 6(c)). At the same time, knockdown of PCAT1 effectively suppressed the size of subcutaneous tumors (Figure 6(d)), and nodules formed on the liver were also significantly reduced (Figure 6(e)).

## 4. Discussion

Studies have reported that colorectal cancer has a high mortality rate and cancer metastasis is an important reason for the high mortality rate [33]. Circulating tumor cells and tumor-derived exosomes are of great significance to tumor progression, and their role in colon cancer remains to be further studied. This study is based on the influence of colorectal cancer exosome lncRNA PCAT1 on the biological behavior of circulating tumor cells. The study finally found that the lncRNA PCAT1 derived from colorectal cancer acts as the ceRNA of miR-329-3p to regulate Netrin-1 in CTCs to promote the EMT process and affect the liver metastasis of colorectal cancer.

An exosome is a vesicle with a diameter of 40-150 nm that can contain a variety of different substances, such as noncoding RNA, DNA, or protein [34]. There have been many studies on inclusions in exosomes. Among these studies, microRNA is a relatively common type in exosomal research [35]; in addition, researchers have also paid attention to the role of a new molecular type, that is, the role of lncRNAs in exosomes [36]. Studies have shown that lncRNA PCAT-1 expression is significantly upregulated in CRC cells and tissues [37, 38], as well as in exosomes extracted from serum of lung cancer patients [39]. Our study also found abnormal expression of PCAT-1 in CRC cells exosomes. In this study, we extracted exosomes from NCM460 cells and the primary colorectal cancer cell lines HCT116 and SW480 and found that the content of exosomes was different and the expression of the long noncoding RNA PCAT1 was abnormally high in colorectal cancer cell lines. lncRNA PCAT1 has been reported to be able to promote the proliferation of prostate cancer cells by binding to the cMyc protein [40]. Our study also found that PCAT1 can promote the proliferation and migration of circulating tumor cells.

The survival of tumor cells escaping from the primary tumor into the bloodstream will lead to subsequent tumor metastasis, so circulating tumor cells have important indicative significance in disease detection [41]. The level of CTCs in peripheral blood is found to be related to a reduction in progression-free survival and overall survival [42, 43]. The characteristics of CTCs in self-renewal and cell differentiation play a role in promoting tumor progression [44, 45]. At the same time, studies have shown that EMT is a cause of tumor cell shedding to form CTCs, so CTCs can exist in the EMT phenotype [46, 47]. Liver metastasis of colorectal cancer is associated with many factors, and EMT may be a key factor, which is manifested mainly in the loss of intercellular adhesion of epithelial cells and the acquisition of mesenchymal properties, thereby promoting tumor invasion and metastasis [48]. EMT is regulated by a variety of proteins, such as the transmembrane protein E-cadherin, which plays an important role in maintaining the polarity and stability of epithelial cells. In addition, the expression of E-cadherin is associated with lymph node metastasis or vascular infiltration [49]. A decrease in E-cadherin levels is often accompanied by the upregulation of N-cadherin expression levels. N-Cadherin is an adhesion molecule that promotes tumorigenesis by enhancing the signaling activity of fibroblast growth factor receptor [50]. Vimentin is also involved in the development of EMT. Vimentin interacts with keratin-KRT14, and Vim gene knockout disrupts the growth and migration of keratinocyte colonies, thereby inhibiting the development of EMT [51]. Zhang et al. [52] observed that after PCAT-1 overexpression, the protein levels of N-cadherin and vimentin were upregulated, and the protein levels of E-cadherin were downregulated, while knockdown of PCAT-1 reversed the above phenomenon. This suggests that PCAT1 is associated with EMT. The same phenomenon was observed in our study, and exosomal PCAT1 was found to be able to significantly reduce the expression of E-cadherin in CTCs and simultaneously promote the upregulation of N-cadherin and vimentin expression, which most truly promotes the occurrence of the EMT process.

The process of EMT can also be regulated by miRNAs. For example, in colorectal cancer with liver metastasis, miR-31-5p may inhibit and support EMT by upregulating c-MET [53], and the downregulation of miR-200 in colorectal cancer increases the expression of ZEB1, thereby promoting EMT [54]. In this study, the overexpression of miR-329-3p was found to also significantly inhibit the occurrence of EMT. Studies have shown that elevation of Netrin-1 induced the typical morphological changes of EMT and increases the expression of EMT markers [25]. Our study found the same effect of Netrin-1, that is, overexpression of Netrin-1 could effectively reverse the inhibitory effect of knockdown of PCAT1 on EMT. Meanwhile, we also found that miR-329-3p negatively regulates the expression of Netrin-1.

## 5. Conclusions

This study found that lncRNA PCAT1 was abnormally highly expressed in exosomes of colorectal cancer cells and determined that PCAT1 can promote the occurrence of EMT in circulating tumor cells by regulating the miR-329-3p/netrin-1 axis and ultimately promote colorectal cancer liver metastasis progression, helping us better understand the mechanism of long noncoding RNAs in the progression of colorectal cancer and providing new ideas for colorectal cancer targeted therapy.

## Data Availability

The datasets used and/or analyzed during the current study are available from the corresponding author upon reasonable request.
